# An Improved Method of Pose Estimation for Lighthouse Base Station Extension

**DOI:** 10.3390/s17102411

**Published:** 2017-10-22

**Authors:** Yi Yang, Dongdong Weng, Dong Li, Hang Xun

**Affiliations:** 1 School of Optoelectronics, Beijing Institute of Technology (BIT) No. 5 Yard, Zhongguancun South Street Haidian District, Beijing 100081, China; smile_yangyi@163.com (Y.Y.); fma1995@163.com (H.X.); 2 Institute of Software Chinese Academy of Sciences, No. 4 South Fourth Street, Zhongguancun, Haidian District, Beijing 100190, China; drli@bit.edu.cn

**Keywords:** pose estimation, base station extension, indoor positioning, Lighthouse, infrared sensor, virtual reality (VR)

## Abstract

In 2015, HTC and Valve launched a virtual reality headset empowered with Lighthouse, the cutting-edge space positioning technology. Although Lighthouse is superior in terms of accuracy, latency and refresh rate, its algorithms do not support base station expansion, and is flawed concerning occlusion in moving targets, that is, it is unable to calculate their poses with a small set of sensors, resulting in the loss of optical tracking data. In view of these problems, this paper proposes an improved pose estimation algorithm for cases where occlusion is involved. Our algorithm calculates the pose of a given object with a unified dataset comprising of inputs from sensors recognized by all base stations, as long as three or more sensors detect a signal in total, no matter from which base station. To verify our algorithm, HTC official base stations and autonomous developed receivers are used for prototyping. The experiment result shows that our pose calculation algorithm can achieve precise positioning when a few sensors detect the signal.

## 1. Introduction

The main task of a tracking system is to detect the spatial poses of objects in three degrees of freedom and six degrees of freedom. In virtual reality systems, a precise and stable tracking system with low latency plays a significant part in creating a truly immersive experience. In vision-based robot systems, accurate and reliable positioning is one of the most fundamental and crucial functions. The cost of camera-based optical tracking systems increases exponentially as the tracking area expands. Thus, economy and stability play pivotal roles in tracking systems.

Lighthouse offers a business indoor tracking solution featuring high precision, low latency and low cost. To solve the problem of tracking objects in the occlusion environment, Lighthouse installs 22 infrared diodes on each handle to receive signals (we regard each diode as a point). Pose estimation is unavailable when the number of points which has detected signal is less than 5. Besides, the Lighthouse does not support base station expansion. This leads to three problems, the first being that the tracked object must reach a certain size. Second, when people acting in the working area, the occlusion problem will be inevitable, so some optical data will be lost in this situation, destabilizing the system. Third, the tracking area cannot be expanded. 

Most of existing pose estimation methods estimate the camera pose from n 3D-to-2D point correspondences. If there exists more than six non-coplanar points in the space, the problem can be easily solved using the well-known direct linear transform (DLT) [1]. When the rank of a matrix is less than 12, the solution of PnP problem is equivalent to the nonlinear problem. There are two solutions: closed-form methods [2,3,4] and iterative optimization methods such as [5,6,7]. The EPnP algorithm is a non-iterative solution for the PnP problem [8], which is used to solve the pose of one camera from n 2D-3D counterparts. The merit of this algorithm is that its computational complexity is O(n). However, it is applicable only when n ≥ 4. Specialized solutions include those designed to solve the P3P problem [2,9,10,11], which are under the constraint of one calibrated camera. It is obviously not applicable for the case where the two base stations recognize n (n ≥ 3) points in total.

Our goal is looking for a pose estimation algorithm to unify the four cases in the same framework with known extrinsic parameter matrixes, namely, rotation matrix R, translation matrix T and the intrinsic matrix of base stations. We can transform the problem into a traditional PnP problem, so as to solve the six degrees of freedom of the tracked object. This algorithm is also applicable in multi-base stations tracking systems.

This paper proposed a unified pose estimation algorithm based on Lighthouse. Through calibration, in the case of known intrinsic parameters matrix of each base station, our algorithm can solve the above four cases correctly. In order to verify the effectiveness of our algorithm, a prototype was built utilizing HTC official base stations and a specifically constructed receiver, whose number of photodiodes and shape of tracker are customizable as per need. The experimental result shows that the tracking system using our algorithm achieves millimeter precision in positioning, and the jitter range is less than 0.4%. A simulation experiment shows that our algorithm is equally applicable to multi- base station tracking systems.

## 2. Materials and Methods 

**Figure 1:**
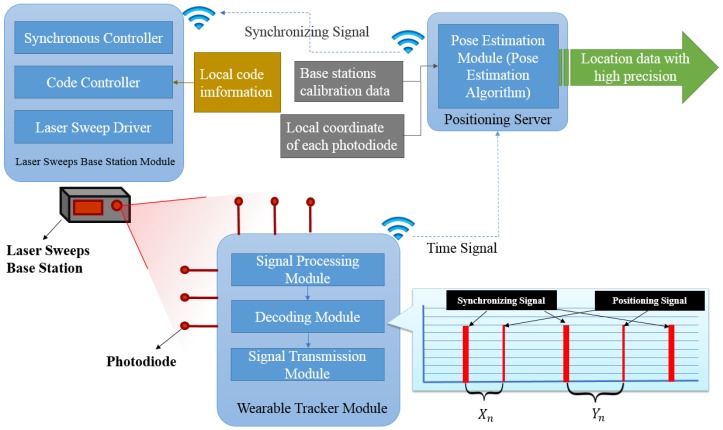
System functional diagram.

Our tracking system consists of three key components: a laser sweeps base stations module, a wearable tracker module and a positioning server, as shown in Figure 1. The function of base station are providing encode control and synchronous control. The local code information is given in advance (the pulse width of synchronizing signal denotes the following line laser from which base station), a LED (Light-emitting diode) array is used for transmitting a sync signal, horizontal and vertical laser sweeps, which are represented as laser sweep driver in Figure 1, and a photodiode, allowing for synchronization between stations when free of cables. The wearable tracker module contains multiple customizable photodiodes with relative position fixed, each of which will pick up signals from base stations and use them to determine the azimuth and elevation angles relative to the base station individually. The photodiode performs preamplification, filtering and quantization of the received laser scanning signal. This is done through the ARM (Advanced RISC Machine) single-chip decoding circuit, which receives the quantization signal to capture, statistics and decoding. The decoded signal is passed through the convert serial port to wireless chip to the positioning server. The input of the positioning server pose estimation algorithm includes the calibration data of each base station, the three-dimensional local coordinate of each photodiode, and the decoded time signal, which is obtained from the wearable tracker module, while the output of positioning server is location data with high precision. 

### 2.1. System Architecture

The proposed tracking system consists of three main sections: two transmitters (base stations); one (or more) tracked object(s), and; a processing unit. The transmitter contains both sync and rotation mechanisms that produce horizontal and vertical laser sweeps. The receiver contains photodiodes, a signal amplifying module, a filtering module and a wireless output module. The processor contains the signal resolver. The tracked object transmits its time data (in millisecond) to a host PC. 

After the laser sweeping signal is received by the photodiode, the front-end circuit performs preamplification, filtering and quantization of the received signal, and the quantized signal received by ARM single-chip decoding circuit is captured, surveyed and decoded. The decoded signal is then transmitted to a PC via wireless connection.

Every tracked object has a unique IP address that they can work independently. An ARM microcontroller is used as processor unit currently, and each chip can load eight receivers at the same time. In the future, we plan to use FPGA (Field－Programmable Gate Array) instead, so that each module could load more photodiodes. By installing customized receivers on different tracked objects, any physical object can be brought into the virtual world. It can be easily attached on various parts of our body, including the head, hand, ankle, and so on, thanks to its small size, flexibility and convenience installation. The most significant feature is that the number and geometry of photosensors can be customized when three or more photodiodes are involved.

### 2.2. Pose Estimation Algorithm

In this paper, the identification process is divided into four cases: (a) base station A identifies three or more points, and B zero; (b) both stations identify three or more points; (c) A or B, and not both, identifies three or more points; (d) neither station alone identifies three or more points, but put together, more than three points are identified. 

**Figure 2:**
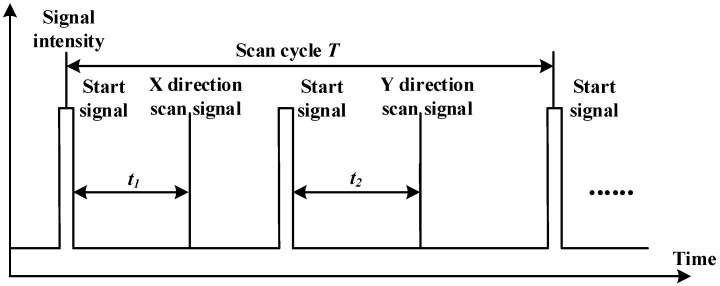
Sample pulse train received by a photodiode on a tracked object. Distance between pulses are not to scale.

The goal of our study is to develop a novel method which can estimate the pose of tracked objects in 3D with 2D coordinates with respect to different base stations. We treat each base station as a pin-hole camera model. A representation of the signal or pulse train received by a photodiode from a base station is shown in Figure 2.

A sync blinker pulse is emitted by an infrared LED array which is built into the transmitter at the beginning of a period. Each photodiode will pick up the signal simultaneously, and followed by sync signal, the vertical laser will scan line swept by rotor 1 in the scan volume, used to measure the interval between the sync signal and the vertical scanning signal, denoted by $t_{1}$ in Figure 2. After rotator 1 rotates 180 degrees, the infrared LED array will blink again as the second sync signal. Then the horizontal laser line swept by rotor 2 in the scan volume will be used to obtain the time interval between the second sync signal and the horizontal signal, denoted as $t_{2}$ in Figure 2. Suppose the rotating speed of both rotors is ω. The azimuth and elevation angles for each photodiode can be calculated as follows:

(1) $$\{\begin{array}{c} \theta_{1}={\omega t}_{1}\\ \theta_{2}={\omega t}_{2} \end{array}$$

The coordinates (x, y) for each sensor in the constellation in a certain base station can be derived as such:

(2) $$\{\begin{array}{c} x=\frac{1}{{\tan\theta }_{1}}\\ y=\frac{1}{{\tan\theta }_{2}} \end{array},$$

The final pixel coordinate (u, v) displayed on the screen can be derived from correlative formulae. For a sensor in the constellation at ${X=[x,\;y,\;z]}^{T}$ in its local coordinate system, the corresponding image coordinates in the emitter is $x_{i}{=[u}_{i},v_{i}{]}^{T},\;i=1,\;2,\;3,\;\ldots \;,\;m$ represents the number of the transmitters, according to the projection imaging principle. Where $\tilde{X}$ and $\tilde{x_{i}}$ are the homogeneous presentations of X and $x_{i}$ respectively, denoted as ${\left[X,\;1\right]}^{T}$ and ${\left[x_{i},\;1\right]}^{T}$. $P_{i}$ is the projection matrix of the i-th emitter, which is obtained in the calibration process. The coordinates of a sensor in the world coordinate system ${\left[X_{w}{,\;Y}_{w}{,\;Z}_{w},\;1\right]}^{T}$. The coordinate of a sensor in the world coordinate system ${\left[X_{w}{,\;Y}_{w}{,\;Z}_{w},\;1\right]}^{T}$ and pixel coordinate ${{[u}_{i}{,\;v}_{i},\;1]}^{T}$ satisfy Equation (3),

(3) $$Z_{c}\times \left[\begin{array}{c} u_{i}\\ v_{i}\\ 1 \end{array}\right]=K_{i}\times \left[\begin{array}{cc} R_{i} & T_{i} \end{array}\right]\times {\left[X_{w}{,\;Y}_{w}{,\;Z}_{w},\;1\right]}^{T}$$

where $Z_{c}$ is a constant, $K_{i}$ is the intrinsic parameter matrix of i-th emitter, $R_{i}$ and $T_{i}$ denote the rotation matrix and translation matrix of the i-th emitter, and $K_{i}$, $R_{i}$ and $T_{i}$ are all known by calibration. Solving the pose of the tracked object simply involves solving R and T. Thus, we can obtain

(4) $$Z_{c}K_{i}^{-1}\left[\begin{array}{c} u_{i}\\ v_{i}\\ 1 \end{array}\right]=\left[\begin{array}{cc} R_{i} & T_{i} \end{array}\right]\left[\begin{array}{cc} R & T\\ 0^{T} & 1 \end{array}\right]{\left[X_{R}{,\;Y}_{R}{,\;Z}_{R},\;1\right]}^{T}$$

Through simplifying, we get the following form:

(5) $$Z_{c}\left[\begin{array}{c} u_{i}^{\prime }\\ v_{i}^{\prime }\\ 1 \end{array}\right]\;=\;P_{i}\;M\;{\left[X_{R}{,\;Y}_{R}{,\;Z}_{R},\;1\right]}^{T}=\left[\begin{array}{c} P_{1}^{T}\\ P_{2}^{T}\\ P_{3}^{T} \end{array}\right]\left[M_{1}{,\;M}_{2}{,\;M}_{3}{,\;M}_{4}\right]{\left[X_{R}{,\;Y}_{R}{,\;Z}_{R},\;1\right]}^{T}=\left[\begin{array}{c} P_{1}^{T}M_{1}\\ P_{2}^{T}M_{1}\\ P_{3}^{T}M_{1} \end{array}\;\begin{array}{c} P_{1}^{T}M_{2}\\ P_{2}^{T}M_{2}\\ P_{3}^{T}M_{2} \end{array}\;\begin{array}{c} P_{1}^{T}M_{3}\\ P_{2}^{T}M_{3}\\ P_{3}^{T}M_{3} \end{array}\;\begin{array}{c} P_{1}^{T}M_{4}\\ P_{2}^{T}M_{4}\\ P_{3}^{T}M_{4} \end{array}\right]{\left[X_{R}{,\;Y}_{R}{,\;Z}_{R},\;1\right]}^{T},$$

For $K_{i}$ is a known matrix, $K_{i}^{-1}$ multiplied by $\left[\begin{array}{c} u_{i}\\ v_{i}\\ 1 \end{array}\right]$ to get $\left[\begin{array}{c} u_{i}^{\prime }\\ v_{i}^{\prime }\\ 1 \end{array}\right]$. Where matrix $P_{i}$ represents $\left[\begin{array}{cc} R_{i} & T_{i} \end{array}\right]$, matrix **M** represents $\left[\begin{array}{cc} R & T\\ 0^{T} & 1 \end{array}\right]$, $P_{1}^{T}$ denotes the first row of $P_{i}$, $M_{1}$ denotes the first column of **M**, and so on, for each parameter. Eliminating $Z_{c}$ in the first two rows with the third row, and we get

(6) $$\{\begin{array}{c} \left(P_{3}^{T}u_{i}^{\prime }-P_{1}^{T}\right)\left(M_{1}X_{R}+M_{2}Y_{R}+M_{3}Z_{R}+M_{4}\right)=0\\ \left(P_{3}^{T}v_{i}^{\prime }-P_{2}^{T}\right)\left(M_{1}X_{R}+M_{2}Y_{R}+M_{3}Z_{R}+M_{4}\right)=0 \end{array},$$

Now we denote $\left(P_{3}^{T}u_{i}^{\prime }-P_{1}^{T}\right)$ and $\left(P_{3}^{T}v_{i}^{\prime }-P_{2}^{T}\right)$ as $C_{i}$ and $D_{i}$, respectively. Both $C_{i}$ and $D_{i}$ are 1 × 4 matrices. Then the equation above is expressed in the form of non-homogeneous linear equation **AX** = **b**,

(7) $$[\begin{array}{llllllllllll} C_{i}(1)X_{R} & C_{i}(2)X_{R} & C_{i}(3)X_{R} & C_{i}(1)Y_{R} & C_{i}(2)Y_{R} & C_{i}(3)Y_{R} & C_{i}(1)Z_{R} & C_{i}(2)Z_{R} & C_{i}(3)Z_{R} & C_{i}(1) & C_{i}(2) & C_{i}(3)\\ D_{i}(1)X_{R} & D_{i}(2)X_{R} & D_{i}(3)X_{R} & D_{i}(1)Y_{R} & D_{i}(2)Y_{R} & D_{i}(3)Y_{R} & D_{i}(1)Z_{R} & D_{i}(2)Z_{R} & D_{i}(3)Z_{R} & D_{i}(1) & D_{i}(2) & D_{i}(3) \end{array}]\times {\left[r_{11}{\;r}_{21}{\;r}_{31}{\;r}_{12}{\;r}_{22}{\;r}_{32}{\;r}_{13}{\;r}_{23}{\;r}_{33}{\;t}_{1}{\;t}_{2}{\;t}_{3}\right]}^{T}=\left[\begin{array}{c} C_{i}\left(4\right)\\ D_{i}\left(4\right) \end{array}\right]$$

where $C_{i}\left(j\right)$ denotes the j-th (j = 1, 2, 3, 4) element of $C_{i}$, $D_{i}\left(j\right)$ denotes the j-th (j = 1, 2, 3, 4) element of $D_{i}$, and $t_{j}$ denotes the j-th (j = 1, 2, 3) element of **T**. For each set of $x_{i}$ and $X_{R}$, two independent equations can be obtained. We thus get 2N equations from N points, leaving 12 unknowns in the equation. Substituting points identified by all emitters into the above equation. When the rank of **A** matrix is equal to 12, the equation is linear, which can be formulated as a least squares problem. However, in order to make our algorithm can work under all circumstances, in this paper, the iterative optimization method is used to solve the derived model. The effective method is selected to solve this problem, which is an optional parameter in MATLAB optimization toolbox. The constraints we used include the following:

(8) $$\{\begin{array}{c} r_{11}^{2}+r_{21}^{2}+r_{31}^{2}=1\\ r_{11}r_{21}+r_{21}r_{22}+r_{31}r_{32}=0\\ r_{12}^{2}+r_{22}^{2}+r_{32}^{2}=1\\ r_{11}r_{13}+r_{21}r_{23}+r_{31}r_{33}=0\\ r_{13}^{2}+r_{23}^{2}+r_{33}^{2}=1\\ r_{12}r_{13}+r_{22}r_{23}+r_{32}r_{33}=0 \end{array}$$

We can get the optimal solution by satisfying the minimum mean square error of the objective function.

### 2.3. Hardware Design

The architecture described in Section 2 is implemented into a functional prototype affordable for general consumers. The implementation is designed to be modular, so we can evaluate each part independently. In general, the whole implementation consists of three different circuit boards (one for pre-signal processing, one for signal decoding, one for serial port to Wi-Fi) and two applications (tracked object firmware and server software).

**Figure 3:**
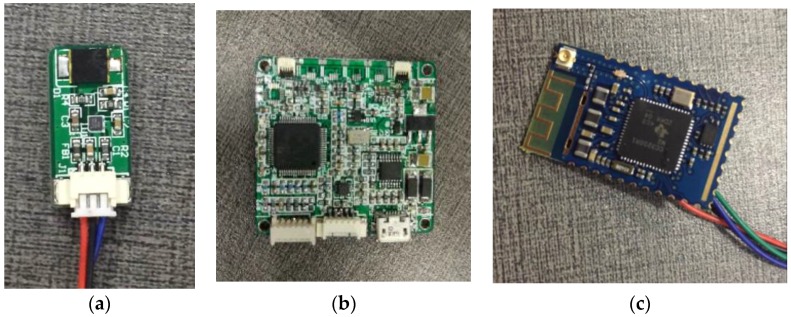
Hardware prototype. (**a**) Prototype of receiver; (**b**) Prototype of processing unit; (**c**) Convert Serial port to Wi-Fi.

**Figure 4:**
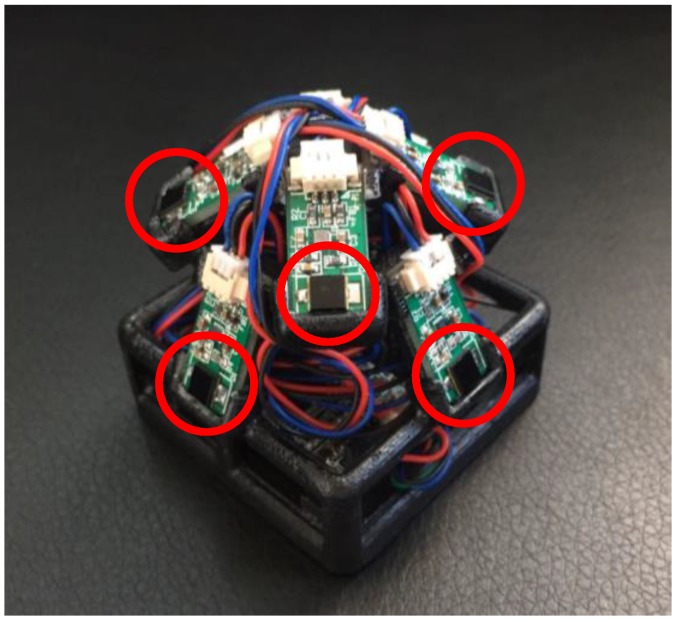
Tracked object consisting of three photodiode sensor boards. The photodiodes are circled in red.

For the tracked object, each receiver unit is designed to hold one photodiode along with its analog front-end circuit, as shown in Figure 3a. VISHAY TEMD5110X01 photodiodes (VISHAY, CA, USA) and Triad TS3633 signal conversion chip (Triad, San Francisco, Palm Harbor, FL, USA) are used featuring invulnerability to visible light interference, high spectral sensitivity and low response time. Its response wavelength is concentrated between 790 nm and 1050 nm, which largely overlaps with the laser emitted by the base station. Each processing module (Figure 3b) can load eight photodiodes. The core processing unit of each processing module circuit use ST STM32 SCM (ST, Geneva, Switzerland), a 168 MHz processor. Figure 3c is the prototype of serial port to Wi-Fi module, and the core component of this module is a TI CC3200 serial (TI, Dallas, TX, USA) to Wi-Fi chip. Of course, the installation of the tracker can be adapted for various scenarios. In this paper, the final installation is shown in Figure 4.

## 3. Results and Discussion

**Figure 5:**
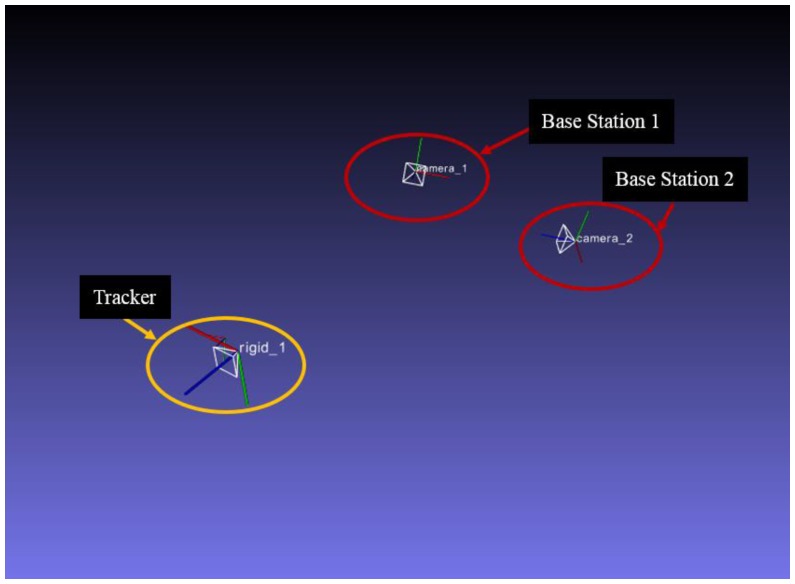
Motion of the tracked object updates the 3D representation in real time.

**Figure 6:**
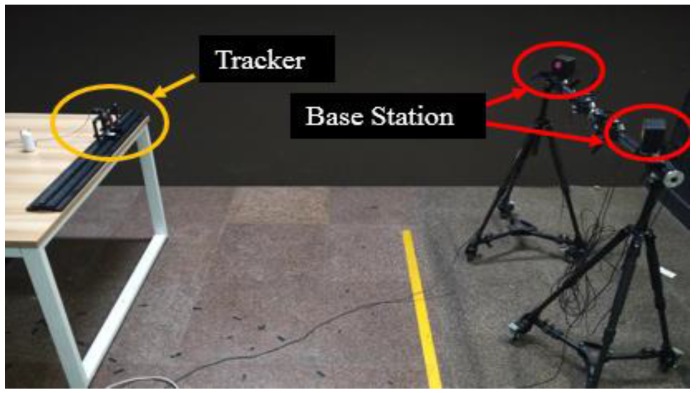
Precision test experimental environment.

In order to evaluate our system, the frame rate was measured first. The hardware frame rate is 272 Hz and the system frame rate is 70 Hz. The pose of tracked object is displayed in Figure 5. The experimental device is shown in Figure 6, which is the setting of our precision test, jitter test and latency test in this environment. The result of these experiments will be given in Section 3.1.1 and Section 3.1.2.

### 3.1. Precision Measurement

#### 3.1.1. Positioning Accuracy

The equipment of our system includes a slide guide, two sliders, a charge pal (which supplies electricity to the tracked object), a tracked object and two HTC Vive base stations. The experimental environment is shown in Figure 6. Three distances in space (1 m, 3 m, 5 m from the base stations) are specified for the test, then in each distance we test the precision along different directions (*x*-axis, *y*-axis, *z*-axis) of the world coordinate. The tracker is installed on the slider, which is moved from 15 cm of the slide guide to 65 cm (step, 2.5 cm), and we pin down the origin of the world coordinate when the tracker is laid on 15 cm. After the tracker is fixed on each position, 1000 valid sets of the tracker’s center coordinates in a virtual environment are collected. Thus, we get 21 × 1000 datasets for each direction/distance. We can obtain the displacement distance from the data of the latter position and the corresponding data of its previous position. We define $\left|X_{u}-\hat{X_{u}}\right|$ as the absolute error, $\hat{X_{u}}$ is 25 here, and the unit is mm.

**Figure 7:**
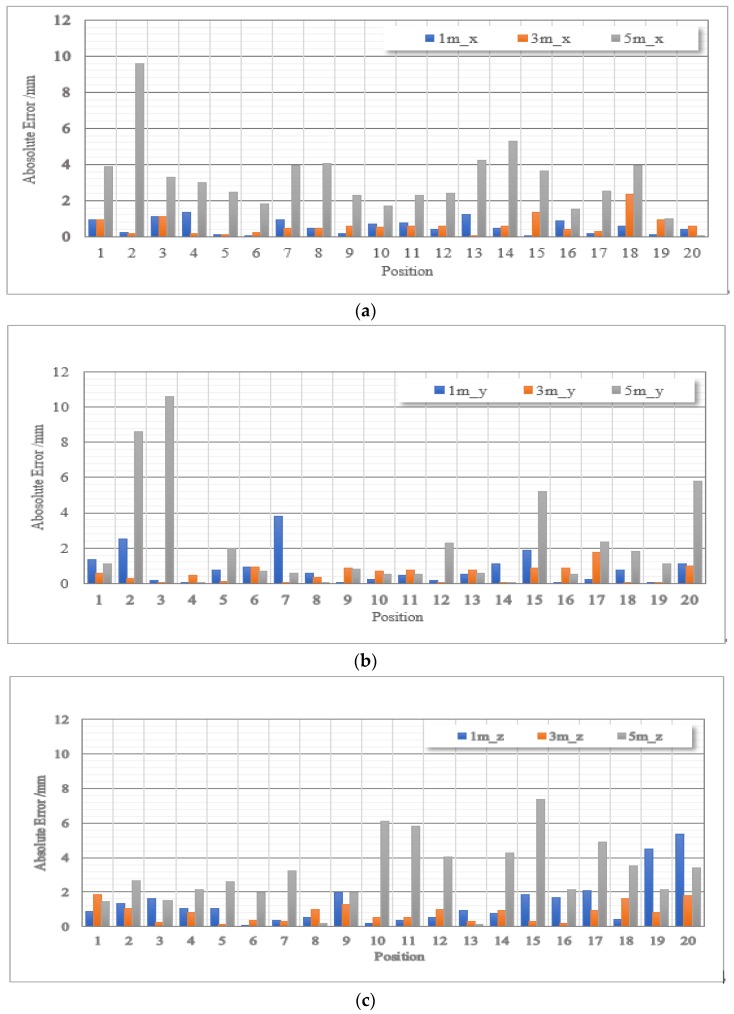
Average range error and at different distances (1 m, 3 m and 5 m). The blue bar refers to the result obtained when the tracker moves along the *x*-axis; the orange bar denotes to the result obtained along *y*-axis and the gray refers to the result obtained along the *z*-axis. (**a**) Average range error at 1 m away from the base stations; (**b**) Average range error at 3 m away from the base stations; (**c**) Average range error at 1 m away from the base stations.

The experimental results are shown in Figure 7. Among these figures, the blue, orange and gray bar represent the result of the tracker in 1 m, 3 m, and 5 m, respectively. The vertical axis means the precision; the unit is mm, and the abscissa axis denotes the 21 positions in the moving process. More specific results was given in Table 1. There may exist partially occlusion during our test, but we could also see the result is quiet stabilize.

It can be seen in Figure 7 that the positional precision of our tracking system is within millimeter level, and the average positioning precision is 1.4653 mm. Most noises are primarily due to mechanical vibrations and inconstant rotational speed of the rotors, which are not arranged perfectly symmetrical. Calibration, measurement and environment factors also contribute to errors. The maximal error is observed in the distance of 5 m from the base stations, reaching 10.6 mm.

#### 3.1.2. Angel Accuracy

**Figure 8:**
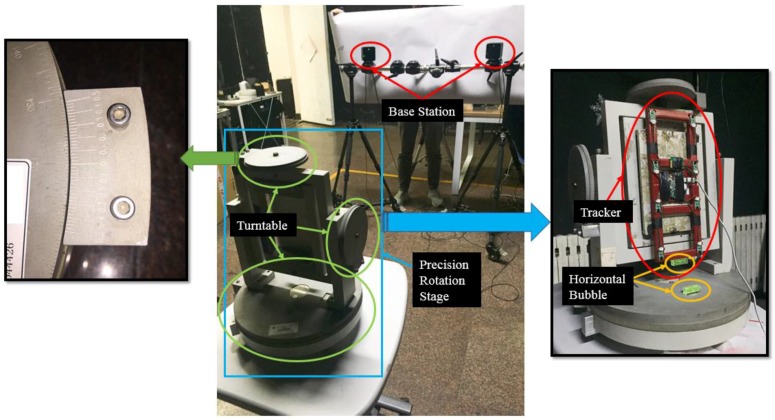
Measuring equipment of angular accuracy.

In order to test the angel accuracy of our algorithm, we stick tracker on a three-axis turntable. There is a built-in bubble on the tracker, so this guarantee the axis of tracker horizontal. In this way, we ensure that the tracker approximately rotates around the only axis. Our angle-measurement system is shown in Figure 8. Three distances in space (1 m, 3 m, 5 m from the base stations) are specified for the test, then in each distance we test the angle precision along different axis (*x*-axis, *y*-axis, *z*-axis) of the world coordinate pivoted. We rotated the tracker from −65° of the rotary stage to 65° (step, 5°), and we pin down the origin of the world coordinate when the tracker is laid on 0°. After the tracker is fixed on each position, 100 valid sets of tracker’s rotation matrix in virtual environment are collected and took average. Thus, we get 25 datasets for each spindle/distance. We use the method in the code of [12] to calculate the tracker’s corner relative to the previous position. The experimental results are shown in Table 2.

It can be seen in Table 2 that the angular precision of our algorithm is within minute level, and the average angle precision is 0.3199°. Most errors are primarily due to mechanical vibrations and inconstant rotational speed of the rotors, which are not arranged perfectly symmetrical. Calibration, measurement and environment factors also contribute to errors. The stability of our system is inversely proportional to distance.

### 3.2. Jitter Measurement

The dataset used in the jitter test is acquired in the same way as the precision test, only processed differently. For the 21 × 1000 groups of data, after getting rid of outliers (<0.5%), Figure 8 shows the scatter point protraction in MATLAB.

**Figure 9:**
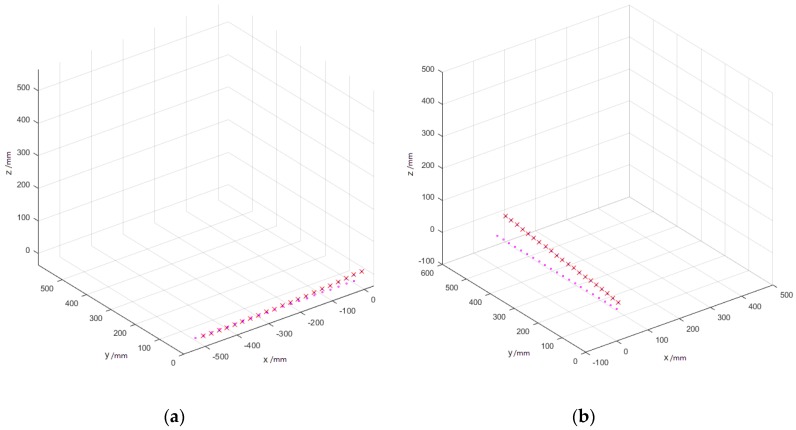

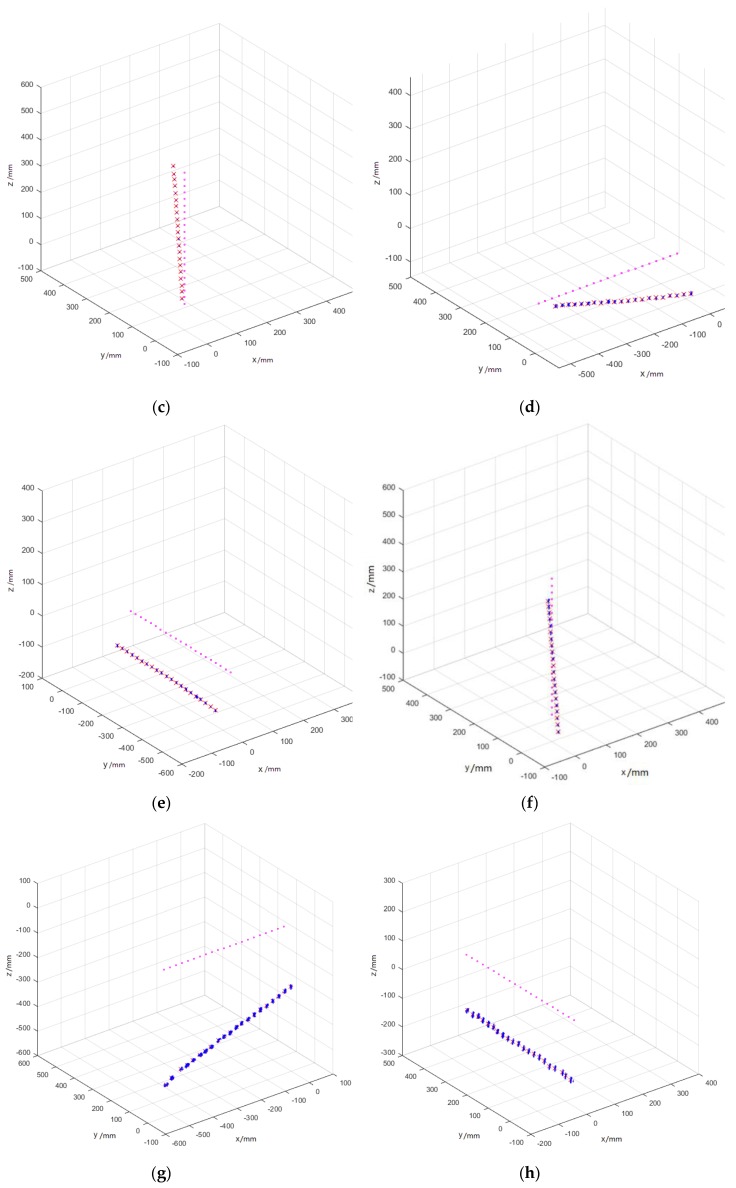

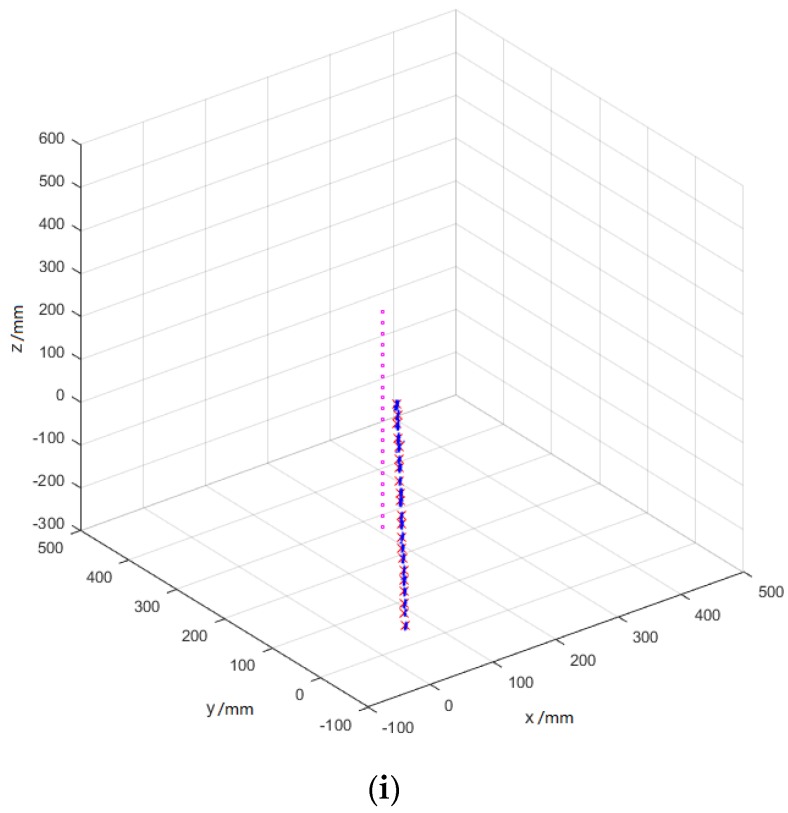
The jitter test results at different distances along each axis. (**a**) 1 m away, along the *x*-axis; (**b**) 1 meter away, along the *y*-axis; (**c**) 1 m away, along the z-axis; (**d**) 3 m away, along the *x*-axis; (**e**) 3 m away, along the *y*-axis; (**f**) 3 m away, along the *z*-axis; (**g**) 5 m away, along the *x*-axis; (**h**) 5 m away, along the *y*-axis; (**i**) 5 m away, along the *z*-axis.

In Figure 9, the blue dots represent the three-dimensional positions in the virtual environment. The red crosses refer to the convex centers of each of the 1000 points. The pink circles denote the ground truth. The size of the blue diffuse spot represents the degree of jitter at this position. As the figure demonstrates, the movement trend of points is consistent with the ground truth, but there exist a certain angle or translation. The further the distance between the tracker and base station, the more obvious the deviation is. The error comes mainly from two sources. One is the systematic jitter, and the other is errors in the process of obtaining world coordinates by taking only one picture. The experimental results show that the jitter of our system is less than 0.28773% at 1 m away from the base stations. The jitter at 3 m is less than 0.487537%, and less than 0.397538% at 5 m. Jitter of various combinations of direction and distance is shown in Table 3. The stability of our system is pretty good since the overall jitter is within 0.4%.

### 3.3. Latency Measurement

**Figure 10:**
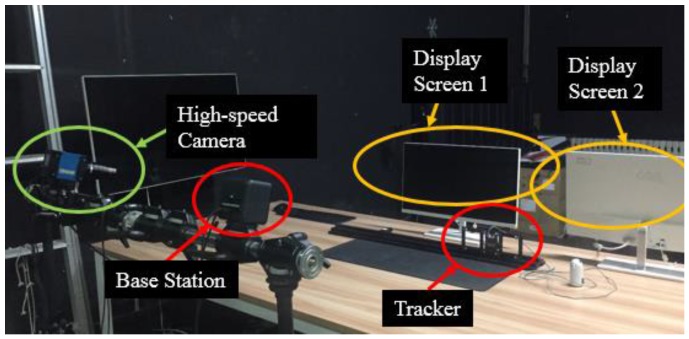
Latency test environment.

The device for latency test includes a 1000-frame high-speed camera, a set of dual-screen computer, an optical slide, two base stations and a tracked object which is shown in Figure 10. We use the high-speed camera to capture the tracker’s movement in both the virtual three-dimensional space and the real world. An indicator light is installed on the tracker so that we can observe the movement of the tracker clearly. When the tracker is quickly dragged on the slide, screen 1 shows the tracker in the virtual three-dimensional space, and screen 2 serves as a monitor. The high-speed camera is used to record the entire process of the tracked object from stationary to swift motion. After the shooting is complete, the video is played frame by frame. The latency of our system is obtained by multiplying the frame rate, which is known, by the difference between the frame number when the tracker begins to move in the virtual space and its counterpart in the real world. The average latency of five measurements is less than 0.047 s.

### 3.4. Simulation Experiment of Multi-Base Station System

To prove that our algorithm is also applicable to multi-base station tracking systems, we have simulated the same process in MATLAB. We assume that there are n base stations (n = 3, 4, 5,…). The parameters of all base stations, such as R matrix and T matrix relative to the world coordinate, are given. We obtain the sample points which has 1000 groups with six points in each group randomly scattered in a 3D space. Each base station is able to identify one or several points in each group. We acquire the pose of the tracked object with our algorithm. When the number of base stations is three, each base station identifies one point, which is the minimum configuration for pose estimation of this algorithm. The calculation is compared with the predicted value, thereby obtaining the calculation error. The statistical result is shown in Table 4. Given that the case where three points are identified by four or five base stations is similar to the case where three points are identified by three base stations, we do not discuss this here. In order to simulate real conditions, we add Gaussian White Noise to the data, mean 0, with different variance (0.8 and 1.2). 

In a wide-range tracking system, a point will be swept by between one to five base stations at the same time, and this experiment covers all possible situations. It can be seen from Table 4 that as the number of stations and points increase, the precision of the pose estimation remains stable. There is little difference between multi-base-station and dual-base-station systems.

## 4. Conclusions

This paper proposes a unified pose estimation algorithm based on Lighthouse. The merit of the algorithm is that it can consolidate data from different base stations in a common framework, making low-cost, large-scale and precise tracking possible. The only constraint is about how many points are identified by all base stations, instead of each base station. The experimental result shows that the positioning precision of tracking system using our algorithm reaches the millimeter level, the angular precision is minute level and the jitter range is less than 0.4%. The simulation experiment shows that our algorithm is equally applicable to multi-base-station tracking systems. While the precision of this system is very high, the results from a low-cost implementation show that there is room for improvement. The future work will focus on improving hardware stability, increasing the number of processing unit ports, as well as expanding base stations to achieve tracking in a broader range.

## Figures and Tables

**Table 1:** The positional performance of our tracking system.

Position	Maximum Error (mm)	Mean Error (mm)	Variance
1 m, along *x*-axis	1.3181	0.5472	0.0515
3 m, along *x*-axis	2.3549	0.6181	1.1526
5 m, along *x*-axis	9.5657	3.1317	26.8151
1 m, along *y*-axis	3.8229	0.8428	0.3132
3 m, along *y*-axis	1.7547	0.5393	2.1978
5 m, along *y*-axis	10.5961	2.2562	19.9662
1 m, along *z*-axis	5.3442	1.3723	0.7553
3 m, along *z*-axis	1.8817	0.8060	22.6941
5 m, along *z*-axis	7.3481	3.0741	62.9562

**Table 2:** The performance of rotation matrix.

Position	Distance (m)	Mean Error (degree (°))	Variance
around *x*-axis	1	0.010938521	0.025690683
	3	0.11624641	1.019411137
	5	0.30455075	2.779377725
around *y*-axis	1	0.04968363	0.137382008
	3	0.355787042	0.442791101
	5	0.746057101	1.787351015
around *z*-axis	1	0.081605419	0.088073508
	3	0.425628327	0.392784724
	5	0.788420395	4.892796488

**Table 3:** Maximum jitter value for different measurement axes in a measurement range of 1 m to 5 m.

Position	Jitter Value (mm)	Jitter scope
1 m, along *x*-axis	1.0747	0.10747%
1 m, along *y*-axis	1.5307	0.15307%
1 m, along *z*-axis	2.2873	0.22873%
3 m, along *x*-axis	7.3874	0.246247%
3 m, along *y*-axis	7.2021	0.24007%
3 m, along *z*-axis	9.7905	0.32635%
5 m, along *x*-axis	19.8356	0.396712%
5 m, along *y*-axis	19.8769	0.397538%
5 m, along *z*-axis	19.3785	0.38757%

**Table 4:** The result of simulation experiment of multi-base stations.

Number of Base Stations	Total Number of Identified Points	Variance of Guassian White Noise	Calculation Error
3	3	0.8	0.041890115
		1.2	0.048899787
	4	0.8	0.00073437
		1.2	0.004322989
	5	0.8	0.010489137
		1.2	0.002100723
	6	0.8	0.006123031
		1.2	0.003626174
4	4	0.8	0.001008683
		1.2	0.068281484
	5	0.8	0.029374229
		1.2	0.171946952
	6	0.8	0.001181789
		1.2	0.002751919
5	5	0.8	0.02913008
		1.2	0.069867259
	6	0.8	0.004555774
		1.2	0.025696456
